# Changes to Master Adaptive Learning in the Pre-Clerkship Phase of Medical School

**DOI:** 10.1007/s40670-026-02794-5

**Published:** 2026-06-20

**Authors:** Luan Lawson, Lisa Domico, Nicole Deiorio, Cana Curtis, Sally A. Santen, Adam Garber, Kacie Lord, Moshe Feldman

**Affiliations:** 1 Virginia Commonwealth University School of Medicine, 1250 E. Marshall St PO 980401, Richmond, VA 23298 USA; 2 Virginia Commonwealth University and American Medical Association, Chicago, IL USA

**Keywords:** Master adaptive learner, Preclerkship curriculum, Self-directed learning and assessment

## Abstract

**Introduction:**

Adaptive expertise or the ability to self-assess, create a learning plan, incorporate self-assessment and feedback, adjust practices with newly learned skills, and address gaps is a core skill in medicine. The Master Adaptive Learner model (MAL) describes a framework to develop adaptive expertise, but studies have not measured adaptive learning during medical school, which limits our ability to design curricular strategies. This study investigated changes in master adaptive learning during the pre-clerkship phase of medical school.

**Materials and Methods:**

This study was a single-cohort within-subject design. Medical students were administered the MAL Instrument at matriculation, end of M1 year and end of M2 year. The MAL instrument included 16 items measured on a 5-point Likert agreement scale measuring 4 factors representing adaptive learning, resilience, curiosity, and motivation. Across and within-subjects ANOVA was used to detect changes in MAL.

**Results:**

There were 174 respondents at matriculation, 81 at the end of M1, and 150 at the end of M2 survey (response rates respectively 94%,41%,89%). 65 students had matched data across all three time points. MAL mean scores increased during the M2 phase for MAL overall and each subfactor. Adaptive learning decreased during the M1 phase but resilience, motivation, and curiosity factors were stable.

**Discussion:**

Medical students MAL scores increased in the M2 phase of the curriculum. The transition into medical school may challenge students’ ability to apply adaptive learning strategies, particularly as they continue developing the metacognitive skills needed to navigate a more complex learning environment.

## Introduction

Modern medicine is evolving at an unprecedented pace. Advances in biomedical science, technology, and health systems are rapidly transforming clinical practice, while the half-life of medical knowledge has continued to shrink. To thrive in this environment, future physicians must not only acquire foundational knowledge, but also develop the capacity to continually assess their learning needs, seek out new knowledge, and adapt their practices [1]. These capabilities—hallmarks of adaptive expertise—are essential for maintaining clinical competence, ensuring patient safety, and contributing to healthcare improvement [2].

This imperative has been recognized across the continuum of medical education. In the United States, the Liaison Committee on Medical Education (LCME) mandates that medical education programs incorporate and evaluate structured opportunities for self-directed learning (SDL), including identifying learning needs, seeking information independently, critically appraising evidence, and integrating feedback (Element 6.3) [3]. At the graduate and continuing medical education levels in the United States, the Accreditation Council for Graduate Medical Education (ACGME) and the American Board of Medical Specialties (ABMS) have established Practice-Based Learning and Improvement (PBLI) as a core competency [4–6]. Similarly, Canada’s CanMEDS framework and the United Kingdom’s General Medical Council’s *Good Medical Practice* embed parallel expectations for lifelong learning, reflection, and quality improvement as integral components of professional competency [7]. Together, these standards underscore the expectation that physicians function as lifelong learners who can reflect on and refine their practice in response to evolving knowledge and patient needs.

Given this continuum-wide emphasis, it is increasingly important to intentionally cultivate and measure these skills during undergraduate medical education, particularly during the preclerkship phase. As students enter medical school, it is important to ensure the development of key learning skills. Foundational behaviors, including goal setting, metacognition, self-assessment, feedback-seeking, and adaptive learning, should not be assumed; rather, they must be explicitly taught, modeled, and assessed. The preclerkship curriculum and the preclinical faculty who design and deliver it, play a foundational role in shaping these adaptive learning behaviors. The learning environment, instructional strategies, assessment approaches, and feedback structures and expected responses are established during the preclerkship phase of medical school send powerful signals to students about how learning is expected to occur. Preclinical faculty are therefore not only responsible for transmitting foundational knowledge, but also for modeling and scaffolding the habits associated with self-directed, reflective, and adaptive learning. Yet, few studies have examined how these behaviors develop over time, particularly during the first years of medical school—a critical period when students are building habits that shape their long-term professional identity. Understanding how Master Adaptive Learner (MAL) behaviors emerge and evolve during the preclerkship phase has direct implications for curricular design, faculty development, and instructional practices in the foundational curriculum.

**Fig. 1:**
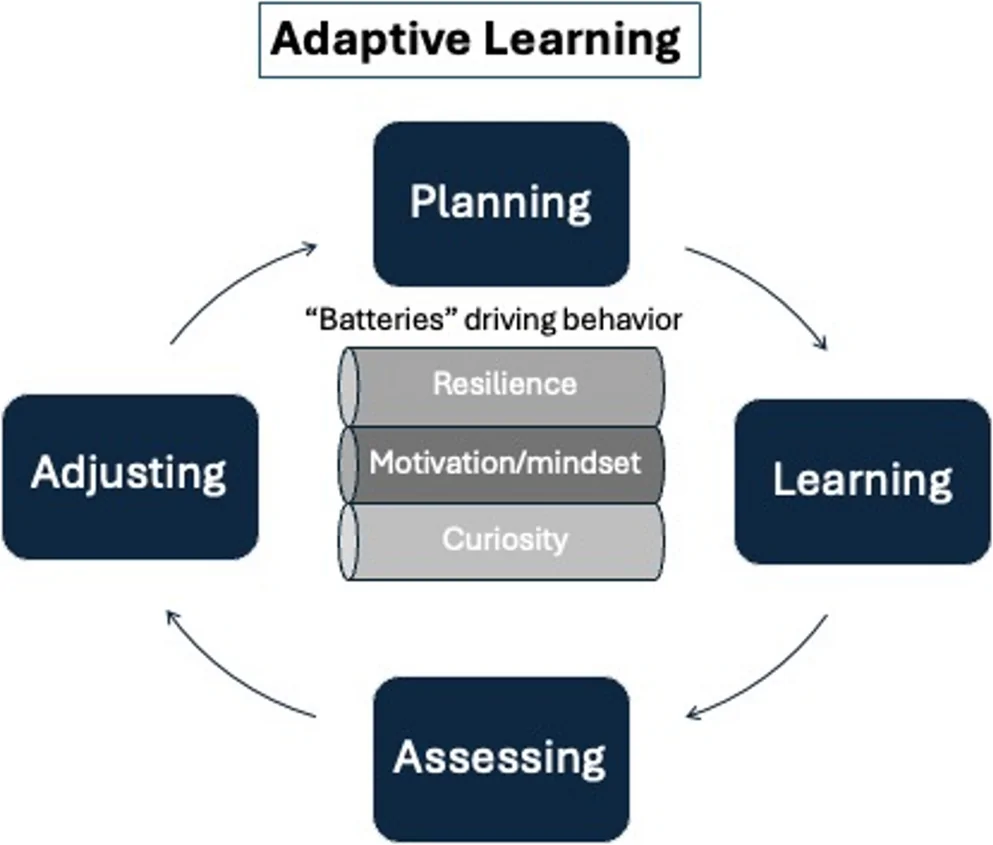
Master adaptive learner model

The MAL conceptual framework offers a developmental model to address this need [2, 8] (Fig. 1). MAL uses elements of the Kolb experiential learning cycle and the Plan-Do-Study-Act healthcare performance improvement process. (concrete experience, reflection, conceptualization, and experimentation) as conceptual frameworks emphasizing goal setting, feedback integration, and continuous performance adjustment in complex healthcare environments [9, 10]. Designed to promote adaptive expertise, the MAL model outlines an iterative process of planning (identifying knowledge gaps), learning (engaging in targeted learning), assessing (self-assessing progress and incorporating feedback), and adjusting(modifying future behavior based on new knowledge and reflection) [11], and shares a foundational conceptualization with Self-Regulated Learning (SRL), a “self-oriented feedback loop” [12]. These master adaptive learning processes are motivated by a set of four ‘batteries’; resilience, mindset, curiosity, and motivation that are theorized to influence the adaptive learning foundational behaviors (e.g. planning, learning, assessing, adjusting). This model and has gained traction in faculty development and residency education. However, little is known about how MAL-related behaviors are expressed or evolve in preclerkship medical students when learners who are navigating the transition from undergraduate to professional education while adjusting to the demands of an accelerated and complex preclerkship curriculum that frequently culminates with demonstrating mastery of foundational knowledge on USMLE Step 1.

This study investigates changes in Master Adaptive Learner behaviors over the preclerkship phase of medical school. By examining how early learners engage in self-directed and adaptive learning over time, we aim to inform curricular strategies that support early development of internal motivation and lifelong learning habits and ensure that graduates are prepared to meet the demands of a rapidly evolving profession.

## Materials & Methods

### Setting

The Virginia Commonwealth University School of Medicine is a large, urban academic medical center in Richmond, Virginia, enrolling 184 medical students per year and offering advanced tertiary and quaternary care through the VCU Medical Center. Medical students participated in a pre-clerkship curriculum phase (i.e. M1 and M2) consisting of 20 months of foundational sciences coursework. The first semester of the M1 year emphasizes basic science knowledge required to understand the structure, function, and pathophysiology of the major organ systems, while the second half of the M1 year and the M2 year are presented using an organ system format that incorporates normal anatomy and physiology before delving into pathophysiology, disease diagnosis, and pharmacology. The M2 curriculum concludes with a period of up to seven weeks of dedicated USMLE Step 1 study time. There is also a parallel longitudinal curriculum that spans the length of the M1 and M2 years and focuses on content that promotes the development of doctoring skills, use of evidence-based medicine, and understanding of the humanistic, ethical, and legal responsibilities of physicians to their patients and society. The foundational curriculum, including the parallel longitudinal curriculum, is graded as pass/fail.

### Study Design

We used a longitudinal study design to evaluate changes in MAL at three time points during the pre-clerkship phase of medical school. A single cohort of medical students were administered the Master Adaptive Learner Instrument [8] at matriculation in July 2023, at the end of the M1 year in May 2024, and at the end of their M2 year in March 2025. Most students had completed their United States Medical Licensing Exam Step 1 exam at the time of M2 survey administration. The study was determined exempt by the Institutional Review Board IRB #HM20014899.

### Measures

The MAL instrument included 16 items measured on a 5-point Likert agreement scale (1 = strongly disagree, 5 = strongly agree) [8]. Items represented four factors of the MAL model including resilience (two items) (e.g. *I tend to bounce back quickly after hard times)*, curiosity (three items) (e.g. *I take every opportunity to learn new things*), motivation/mindset (three items) (e.g. I prefer to engage in learning that is challenging) and adaptive learning behaviors or ‘cogs’ (eight items) (e.g. *I identify the resources needed during the learning process*).

A mean score was calculated for MAL overall using all items and each of the four factors. A repeated measures analysis of variance was used to test for changes in MAL factors between matriculation, end of M1, and end of M2.

Recognizing the subjects were repeated across several administrations, a within subject comparison would provide the strongest inference of individual changes in MAL over time, but also suffers from availability of data at all three time points. Given this, a secondary between subject analysis was conducted to detect any sampling bias in the smaller sample. Paired sample t-tests were used to test for differences in mean MAL scores at each timepoint.

## Results

### Sample

There were 174 respondents to the matriculation survey (response rate = 94%), 81 total respondents to the end of M1 survey (response rate = 41%), and 150 respondents to the end of M2 survey (response rate = 89%). Descriptive statistics for the full sample are provided in Table 1.

**Table 1:** Number of respondents and response rates for MAL overall and each factor

	Matriculation		End M1		End M2	
	*N*	RR	*N*	RR	*N*	RR
MAL Overall (16 items)	172	93%	81	41%	148	80%

### Within-Subject Analysis

There were 65 respondents with matched data at all three time points that were included in the within subjects’ analysis representing a 35% response rate for the total class. Coefficient alpha was greater than 0.84 across all survey MAL factors.

There was a statistically significant change across time points on MAL overall (F = 23.58, df = 2, *p* < 0.001), adaptive learning (F = 11.46, df = 2, *p* < 0.001), resilience (F = 15.96, df = 2, p <,001), (F = 23.58, df = 2, *p* < 0.001), curiosity (F = 11.51, df = 2, *p* < 0.001), and motivation (F = 17.78, df = 2, *p* < 0.001) (Table 2).

**Table 2:** Means and standard deviations for MAL overall and each factor

	Matriculation		End M1		End M2	
	Mean	SD	Mean	SD	Mean	SD
MAL Overall (16 items)	4.09	0.40	4.06	0.48	4.38	0.44
Resilience (2 items)	3.74	0.76	3.67	0.97	4.28	0.64
Curiosity (3 items)	4.08	0.59	4.17	0.65	4.10	0.61
Motivation/Challenge Mindset (3 items)	4.01	0.64	4.00	0.70	4.11	0.61
Adaptive Learning (8 items)	4.22	0.41	4.15	0.46	4.37	0.45

**Fig. 2:**
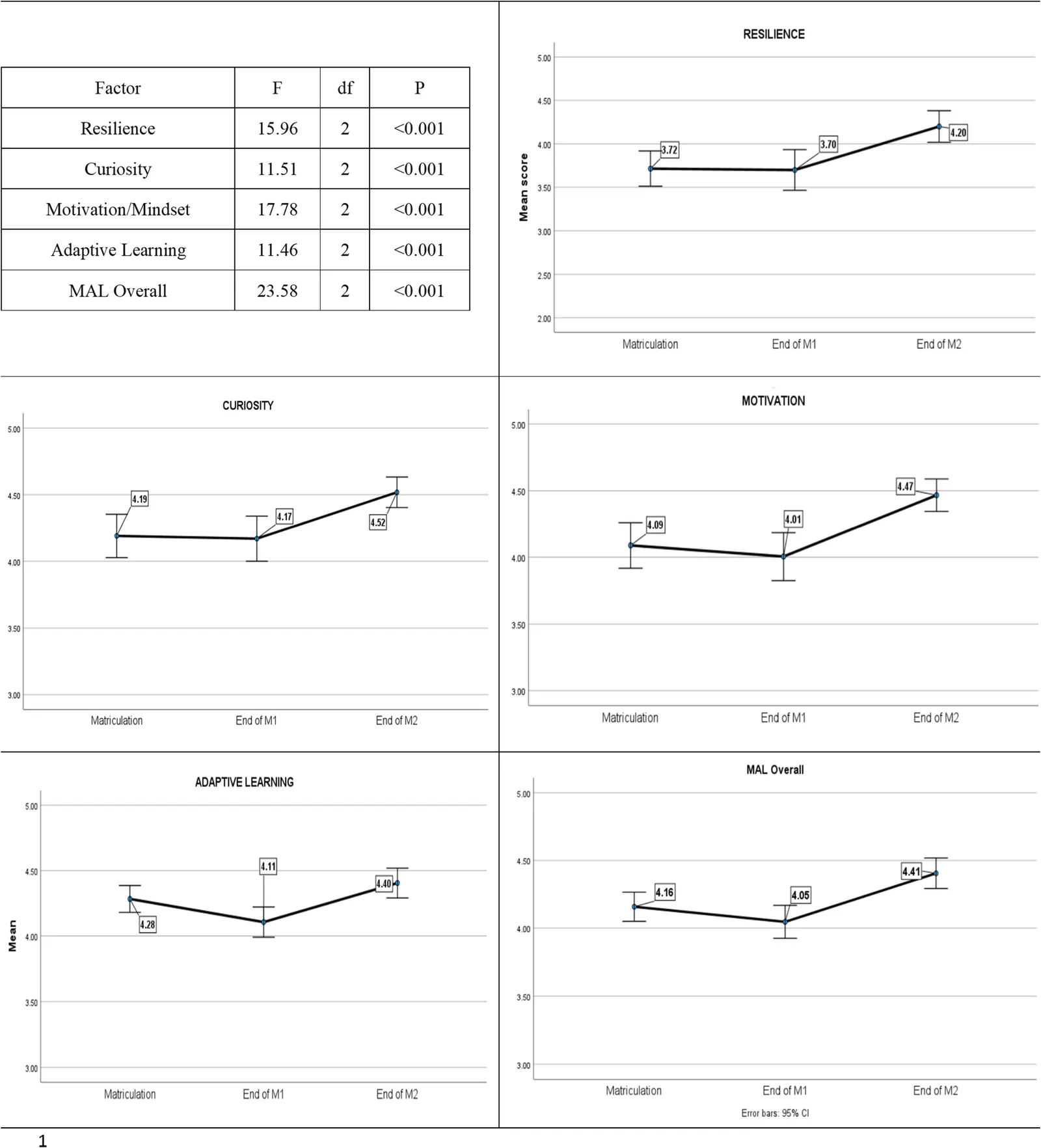
Mean MAL factor scores during the pre-clerkship curriculum. *N* = 65. Error bars 95% confidence interval

There was a statistically significant change across time points on MAL overall (F = 23.58, df = 2, p < 0.001), adaptive learning (F = 11.46, df = 2, p < 0.001), resilience (F = 15.96, df = 2, p < ,001), (F = 23.58, df = 2, p < 0.001), curiosity (F = 11.51, df = 2, p < 0.001), and motivation (F = 17.78, df = 2, p < 0.001). Students responded with higher mean scores on MAL overall (Mean = 4.41, SD = 0.46), resilience (Mean = 4.20, SD = 0.73), curiosity (Mean = 4.52, SD = 0.46), and motivation (Mean = 4.47, SD = 0.49) at the end of M2 compared to end of M1 and matriculation (*p* < 0.001) (Fig. 2). Adaptive learning was higher at the end of M2 (Mean = 4.40, SD = 0.46) compared to the end of M1, but lower at the end of M1 (Mean = 4.11, SD = 0.41) compared to matriculation (Mean = 4.28, SD = 0.41).

### Secondary Between Subject Analysis

A secondary between-subjects analysis was conducted to determine potential sampling bias due to the lower sample size from the within subject analysis. Between subject t-tests showed parallel results to the within subjects with similar differences in effects observed.

## Discussion

This was the first study using the revised MAL instrument. This within subject, repeated measures study showed medical students improved as master adaptive learners during the M2 year of the curriculum. During the M1 year, MAL batteries (i.e. resilience, curiosity, motivation) remained similar to matriculation levels, but mean scores on adaptive learning processes decreased. The transition into medical school may challenge students’ ability to apply adaptive learning strategies, particularly as they continue developing the metacognitive skills and self-awareness needed to navigate a more complex and structured learning environment. A variety of factors likely contribute to the lower adaptive learning process scores observed at the end of the M1 year. These included the largely didactic nature of foundational science instruction, the high cognitive load associated with preclinical content, and the ongoing development of metacognitive awareness and self-regulated learning skills during this early stage of training. Self-determination theory suggests that these factors would lower motivation during this time because all three of its components suffer: autonomy is low due to the highly-prescribed curriculum in the pre-clinical phase; sense of competence may be challenged as they may no longer be the consistent high-achievers they had been in college; and relatedness can suffer as individual performance is emphasized to a greater degree that team performance in this curricular period. While aspects of the pre-clinical curriculum are essential for attaining fundamental knowledge and ensuring attainment of core competencies, they may simultaneously present challenges to the development and application of adaptive learning strategies in the early phases of medical education, particularly during the first year of medical school.

Traditional, lecture-based instruction, typical of first-year undergraduate medical education curricula, offers a structured and efficient way to establish foundational knowledge in disciplines such as biochemistry, physiology, genetics, and immunology. These subjects, inherently complex and delivered within a compressed timeframe, demand a high cognitive load [13, 14]. The structured nature of this learning environment often leaves little room for students to engage in self-directed exploration or to adjust their personal learning strategies based on feedback, limiting opportunities to recognize their own knowledge gaps or develop individualized learning objectives [15]. Consequently, students may prioritize content coverage and efficiency over reflection and adaptability. The cumulative demands of intense coursework, frequent assessments, and a highly structured learning environment highlight the importance of incorporating opportunities for reflection, individualized learning plans, coaching, and self-directed learning to promote resilience and thrive in the preclinical environment [16, 17].

During this period, many students are also unfamiliar with both the content and the clinical vignette-based assessment style typical of medical school exams. This unfamiliarity can make it difficult for them to identify effective study strategies or to recognize and address their own learning needs. While students do receive formative feedback through practice questions and assessments, this feedback tends to focus primarily on content acquisition rather than on promoting metacognitive reflection or the identification of areas for self-improvement. Furthermore, the progression of the preclinical curriculum, from one high-yield subject to the next, leaves limited opportunity for students to revisit previously covered material or to address identified knowledge gaps. Without structured opportunities for reflection, feedback integration, and self-directed learning, these conditions may contribute to lower MAL scores. The cumulative demands of high-intensity coursework, frequent assessments, and limited autonomy in shaping one’s learning trajectory can take a psychological and cognitive toll if not balanced by intentional supports within the curriculum.

By the second year of medical school, however, students are typically better positioned to engage in adaptive learning strategies due to both the cumulative experience gained during the first year and the evolving nature of the medical education curriculum. Students have had a year to navigate the pace and structure of the medical education, build foundational knowledge, and acclimate to assessment formats. They have also received iterative feedback in longitudinal courses such as *Practice of Clinical Medicine* and *Diagnostic Reasoning*, which emphasize critical thinking, clinical application, and self-reflection. These experiences support the development of time management skills and the ability to experiment with different learning strategies, both of which are key components of metacognitive learning as students transition into more clinically based clerkship year [18, 19].

Additionally, during M2 year, students begin to reflect on their preparation for the Step 1 exam. The dedicated study period, up seven weeks at the conclusion of the second year curriculum, is largely self-directed and often initially guided by a faculty coach or advisor. These coaches help students develop study structures based on self-reflection and performance in the preclinical phase of the curriculum. Commercial resources, such as question banks, are widely used and likely promote the use of self-assessment and metacognitive strategies, further reinforcing MAL learning behaviors [20]. These experiences, combined with the cognitive scaffolding established in the M1 year, support a shift toward greater metacognitive engagement and self-directed learning. As a result, the second-year curriculum, by both its design and the learner’s developmental stage, creates more space for the practice of adaptive learning strategies.

Often during the third year, the medical curriculum transitions into a less structured clinical learning environment where adaptive learning is increasingly important due to the need apply of knowledge in ambiguous patient contexts [21]. This cycle of reflection, adjustment, and reassessment is central to metacognitive skill development and is a behavior that continues to be cultivated throughout medical school and into residency training.

Given these findings, curricular changes in the preclinical phase may support earlier development of MAL behaviors by fostering an educational culture that promotes adaptive learning and ensures access to appropriate resources [22]. Incorporating more coaching—focused on self-reflection, metacognitive skills, and fostering a growth mindset—could promote a more supportive environment for adaptive learning [23, 24]. Embedding self-directed learning (SDL) activities earlier in the curriculum may also offer students valuable opportunities to practice these skills while still building foundational knowledge. These strategies represent feasible and impactful adaptations, rather than requiring a complete revision of the curriculum. Additionally, expanding existing active learning strategies could further enhance student engagement with metacognitive and adaptive learning processes, better preparing them for the demands of clinical training and promoting lifelong learning [25].

## Limitations

This study has several limitations. First, this was a single-institution study of one medical school’s preclinical curriculum, which limits generalizability to other institutions with different curricular structures (e.g., problem-based learning). The structured design of the preclinical curriculum may have influenced MAL scores differently than in schools where there may be more emphasis on independent student attainment of foundational knowledge. The study relied on self-reported measures of MAL behaviors that may not accurately reflect the students’ actual adaptive learning behaviors or could be impacted by external factors. Although survey response rates were high at matriculation and at the end of M2, the end-of-M1 response rate was substantially lower (41%), which may introduce nonresponse bias. The M1 survey was administered asynchronously which may explain the lower response rate compared to the M2 survey where students were provided dedicated time to complete. While the between-subject analysis suggested no systematic sampling bias, the possibility remains that students who completed all three surveys differed in meaningful ways (e.g., motivation, study strategies) from those who did not. Finally, the student period concluded with a unique curricular and assessment context. Students were preparing for Step 1 in a pass/fail assessment approach, which may have influenced their learning approaches or their perception of adaptive learning behaviors. The original MAL model theorized 4 batteries, but the factor analysis of the revised instrument found 3 distinct batteries from the items included. This may be due to insufficient items to represent the full model or that the mindset and motivation factors have overlap and are difficult to measure as unique contructs. Additional studies should incorporate complementary approaches such as qualitative analyses or performance assessments and extend across multiple institutions and medical student cohorts to provide a more comprehensive and generalizable understanding of MAL development over time.

## Conclusion

In conclusion, this study suggests that there may be opportunities to incorporate the practice of MAL in the first year of medical school. The overall process of MAL decreased over the M1 year, with the largest decrease in adaptive learning. The M1 curriculum is very structured and based on learning foundational concepts and basic clinical skills. Although data from additional cohorts is necessary, there may be opportunities to integrate additional, meaningful active learning activities and intentional self-directed learning experiences within the first year of medical school. Incorporation of opportunities to explore MAL characteristics and behaviors may enhance medical education and ultimately, improve patient care.

## Data Availability

NA.

## References

[1] Cutrer W, Spickard W, Triola M, Allen B, Spell N, Herrine S, et al. Exploiting the power of information in medical education. Med Teach. 2021;43:S17-24. 10.1080/0142159X.2021.1925234. 34291714

[2] Cutrer W, Russell R, Davidson M, Lomis K. Assessing medical student performance of Entrustable Professional Activities: a mixed methods comparison of Co-Activity and Supervisory Scales. Med Teach. 2020;42(3):325–32. 10.1080/0142159X.2019.1686135. 31714166

[3] Liaison Committee on Medical Education. Data Collection Instrument (DCI) for Full Accreditation Surveys Academic Year 2025-26. Washington, DC: LCME. Accessed April 15. 2026. https://lcme.org/schools/full-survey-documents

[4] American Board of Medical Specialties. Standards for Initial Certification. [Internet]. American Board of Medical SpecialtiesABMS; 2016 [cited 2025 June 30]. Available from: https://www.abms.org/wp-content/uploads/2020/11/abms-standards-for-initial-certification-20160511.pdf https://www.abms.org/wp-content/uploads/2021/05/standards-for-initial-certification.pdf Accessed 30 June 202.

[5] Accreditation Council for Graduate Medical Education (ACGME). The Milestones Guidebook. 2nd ed. 2020.

[6] Frank J, Snell L, Sherbino J. CanMEDS 2015 Physician Competency Framework. Ottawa: Royal College of Physicians and Surgeons of Canada; 2015.

[7] General Medical Council. Good medical practice 2024. [Internet]. General Medical Council, London GMC. 2024. Available from: https://www.gmc-uk.org/professional-standards/the-professional-standards/good-medical-practicehttps://www.gmc-uk.org/cdn/documents/good-medical-practice-2024---english_pdf-102607294.pdfhttps://www.gmc-uk.org/professional-standards/the-professional-standards/good-medical-practice.

[8] Santen SA, Funk A, Feldman M, Ginzburg SB, Ryan MS, Cutrer WB, et al. Measuring the master adaptive learner: development and validity evidence for a revised instrument. Med Teach. 2025. 10.1080/0142159X.2025.2536152. 40755238

[9] Kolb DA. Experiential learning: experience as the source of learning and development. Englewood Cliffs, N.J: Prentice-Hall; 1984. p. 256.

[10] Taylor MJ, McNicholas C, Nicolay C, Darzi A, Bell D, Reed JE. Systematic review of the application of the plan–do–study–act method to improve quality in healthcare. BMJ Qual Saf. 2014;23(4):290–8. 10.1136/bmjqs-2013-001862. PMC3963536 24025320

[11] Cutrer WB, Miller B, Pusic MV, Mejicano G, Mangrulkar RS, Gruppen LD, et al. Fostering the development of master adaptive learners: a conceptual model to guide skill acquisition in medical education. Acad Med. 2017;92(1):70–5. 10.1097/ACM.0000000000001323. 27532867

[12] Zimmerman BJ. Self-regulated learning and academic achievement: an overview. Educ Psychol. 1990;25(1):3–17. 10.1207/s15326985ep2501_2.

[13] Sweller J. Cognitive Load Theory. In: Psychology of Learning and Motivation. Elsevier; 2011. p. 37–76. 10.1016/B978-0-12-387691-1.00002-8.

[14] Leppink J, Van Den Heuvel A. The evolution of cognitive load theory and its application to medical education. Perspect Med Educ. 2015;4(3):119–27. 10.1007/S40037-015-0192-X. 26016429 PMC4456454

[15] Driessen E, Van Tartwijk J. The role of reflection in medical education. Perspect Med Educ. 2012;1(1):17–26. 10.1007/s40037-012-0005-z.

[16] Romanova A, Humphrey-Murto S, Pusic M, Touchie C. Individualized learning plans for active lifelong learning. Acad Med. 2025. 10.1097/ACM.0000000000006049. 40137840

[17] Cutrer WB, Atkinson HG, Friedman E, Deiorio N, Gruppen LD, Dekhtyar M, et al. Exploring the characteristics and context that allow master adaptive learners to thrive. Med Teach. 2018;40(8):791–6. 10.1080/0142159X.2018.1484560. 30033795

[18] Lu J, Liu Y, Liu S, Yan Z, Zhao X, Zhang Y, et al. Machine learning analysis of factors affecting college students’ academic performance. Front Psychol. 2024;15:1447825. 10.3389/fpsyg.2024.1447825. 39764083 PMC11700736

[19] Khalil MK, Williams SE, Hawkins HG. The use of learning and study strategies inventory (LASSI) to investigate differences between low vs high academically performing medical students. Med Sci Educ. 2020;30(1):287–92. 10.1007/s40670-019-00897-w. 34457669 PMC8368407

[20] Verma N, Yui JC, Record JD, Hueppchen NA, Naik RP. The changing landscape of the preclinical medical school curriculum: results from a nationwide survey of United States medical school curriculum deans. Am J Med. 2024;137(2):178-184.e2. 10.1016/j.amjmed.2023.10.021. 37890571

[21] Regan L, Hopson L, Gisondi M, Branzetti J. Learning to learn: a qualitative study to uncover strategies used by master adaptive learners in the planning of learning. Med Teach. 2019;41(11):1252–62. 10.1080/0142159X.2019.1630729. 31287741

[22] Auerbach L, Santen S, Cutrer W, Daniel M, Wilson-Delfosse A, Roberts N. The educators’ experience: learning environments that support the master adaptive learner. Med Teach. 2020;42(11):1270–4. 10.1080/0142159X.2020.1801998. 32755327

[23] Wolff M, Hammoud M, Carney M. Developing master adaptive learners: implementation of a coaching program in graduate medical education. West J Emerg Med. 2023;24(1):71–5. 10.5811/westjem.2022.12.57636. 36735006 PMC9897244

[24] Klig JE, Kettyle WM, Kosowsky JM, Phillips WR Jr., Farrell SE, Hundert EM, et al. A pilot clinical skills coaching program to reimagine remediation: a cohort study. MedEdPublish. 2023;13:29. 10.12688/mep.19621.2. 37674590 PMC10477753

[25] Shorbagi AI. Promoting resilience among medical students using the Wadi framework: a clinical teacher’s perspective. Front Med. 2024;11:1488635. 10.3389/fmed.2024.1488635. PMC11527645 39493710

